# Supplementary figures and images for: Estimating the sample mean and standard deviation from the sample size, median, range and/or interquartile range
Source: BMC Med Res Methodol. 2014 Dec 19;14:135. doi: 10.1186/1471-2288-14-135 (PMC4383202; doi:10.1186/1471-2288-14-135)

**Log-Normal**

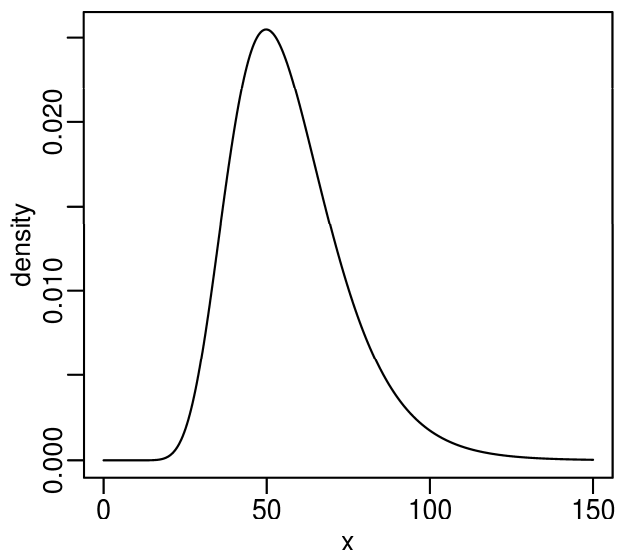

**Beta**

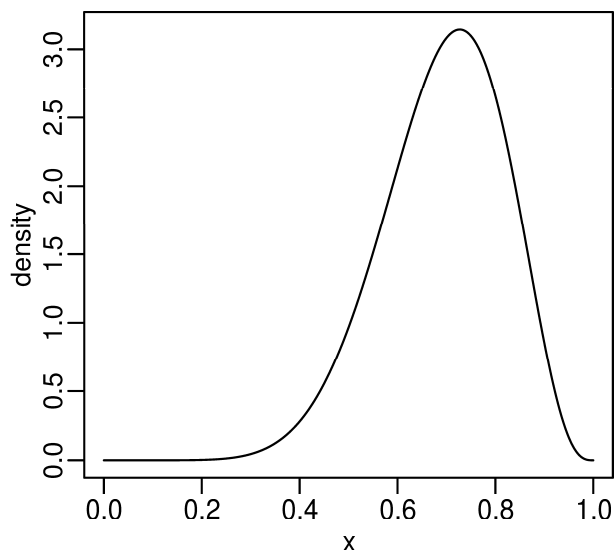

**Exponential**

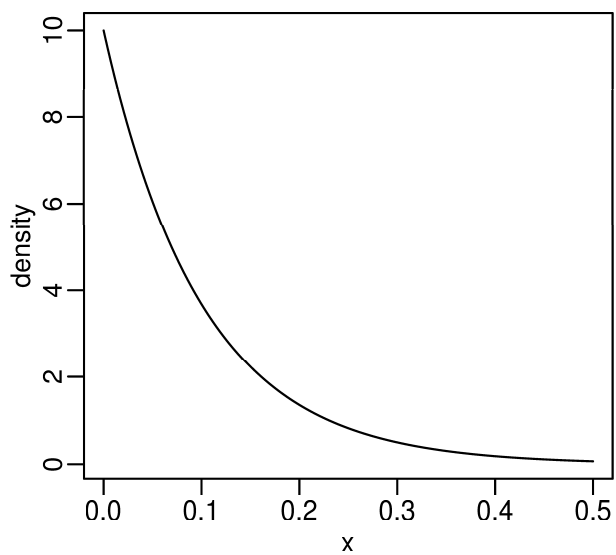

**Weibull**

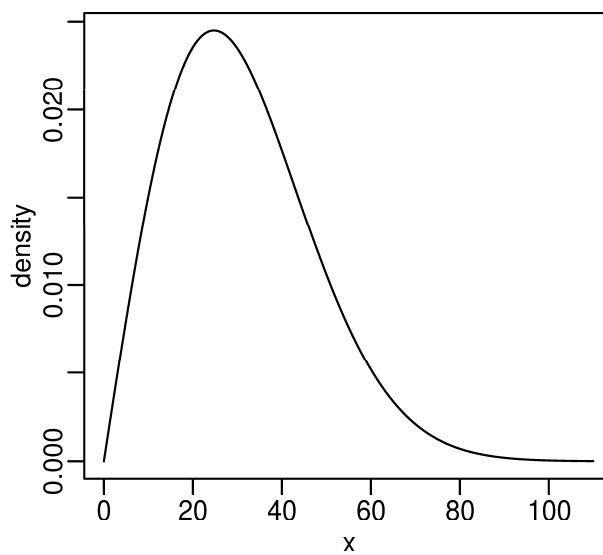

Supplement: Supplementary file 1 — Additional file 1: The plot of each of those distributions in the simulation studies. (PDF 45 KB) [file 12874_2014_1175_MOESM1_ESM.pdf]
